# An International Comparison of Absolute Radiant Power Measurement Capabilities

**DOI:** 10.6028/jres.095.040

**Published:** 1990

**Authors:** Douglas B. Thomas

**Affiliations:** National Institute of Standards and Technology, Gaithersburg, MD 20899

**Keywords:** absolute spectral response, intercomparison, laser power, photodetector, radiant power, radiometry, silicon photodiodes

## Abstract

We report the results of an intercomparison of monochromatic radiant power measurement capabilities recently completed by 11 national laboratories. The intercomparison radiometers, distributed in pairs, included an amplifier with six decades of precision gain and one of two types of silicon photodiode (*pn* or *np*-type construction). Eleven of the laboratories measured the absolute responsivity of the radiometers at 633 nm and nine at 488 nm. The standard deviation of the overall difference was 0.36% at both wavelengths. The agreement between the various participating laboratories and NIST was within the measurement accuracy stated by the participants.

## 1. Introduction

In 1986 an intercomparison of monochromatic radiant power measurements at 633 and 488 nm was undertaken at the request of the Comité Consultatif de Photométrie et Radiométrie (CCPR). The lead laboratory was the National Institute of Standards and Technology (NIST) (formerly the National Bureau of Stanards). The participating laboratories were: Electrotechnical Laboratory (ETL), Japan; Institut National de Métrologie (INM), France; National Institute of Measurements (NIM), Chengdu, People’s Republic of China; CSIRO National Measurement Laboratory (NML), Australia; National Office of Measures (OMH), Hungary; National Physical Laboratory (NPL), United Kingdom; National Physical Research Laboratory (NPRL), South Africa; National Research Council (NRC), Canada; Physikalisch-Technische Bundesanstalt (PTB), Federal Republic of Germany; and Research Institute of Technical Physics of the Hungarian Academy of Sciences (MFKI), Hungary. The intercomparison was organized by E. F. Zalewski (formerly with NIST) and a report on the intercomparison was prepared by him and submitted to the CCPR for publication in the proceedings of the eleventh session of the CCPR [1]. This paper is a subsequent update of the analysis presented in the report to the CCPR and presents a more detailed error analysis of the data and some typographical corrections.

This was the first international comparison of monochromatic radiant power measurements using silicon photodiodes as the transfer standards. A previous intercomparison of laser power measurements [2,3] used thermal detectors as transfer standards, while an intercomparison of (spectrally total) radiant power [4] used incandescent lamps. Recent reports on the behavior of various types of silicon photodiodes under varying environmental conditions [5–8] suggest good measurement stability can be obtained by the appropriate selection of silicon photodiode and use of an improved design of the calibration transfer device.

The intercomparison procedure will be described along with some details of the absolute radiant power measurement procedures employed in each of the participating laboratories.

## 2. Experimental Conditions

The comparison method consisted of each laboratory measuring the absolute spectral response of circulated silicon photodiode radiometers. The participating laboratories measured absolute responsivity at 488 and 633 nm (argon ion and helium-neon laser wavelengths) within the central portion of the photodiode’s active area. NIST, as the central laboratory, measured the responsivity before and after the participating laboratory. In some cases, due to equipment problems, this protocol was not maintained. A detector responsivity comparison is equivalent to comparing each laboratory’s capability of measuring radiant power. This intercomparison has added significance in radiometry arising from the new definition of the candela which enables realization of this unit from a radiant power measurement base [9].

To allow for maximum uniformity of measurement in the participating laboratories, complete radiometers were circulated that included both the photodiode and the signal processing electronics. This precluded any complications in the intercomparison arising from different amplification techniques and possible inaccuracies in gain calibrations. The signal processing electronic circuit consisted of an operational amplifier in a transimpedance configuration (current to voltage amplifier) and a buffer amplifier. The gain settings of the radiometers were variable in six precise decade steps from 0.1 mA/V to 1 nA/V. The absolute uncertainty in the gain was less than 0.1% in the 0.1 mA/V to 100 nA/V range. At the two higher gain settings the absolute uncertainty increased to 0.5 and 1%, respectively.

Stability of the transfer device is an important consideration in any intercomparison. At the time this intercomparison was being planned, it was known that the collection efficiency for minority carriers generated near the oxide-silicon interface in *pn*-silicon photodiodes could be unstable [10,11,12], Since the collection efficiency in the *np* induced-junction type silicon photodiode is immune to changes at the oxide-silicon interface [13],it was decided that photodiodes of this type would also be circulated for the comparison. Circulating both types was deemed necessary because very little evidence concerning the stability of the new *np* type of silicon photodiode existed at that time. The two different silicon photodiodes used were a *pn* type that was manufactured by the EG&G Company^1^ [14], Model UV-444B, and an *np* type incorporating the recently developed induced junction technology that was manufactured by the United Detector Technology Company (UDT) [15], Model UV-100. A total of 16 radiometers were constructed for this intercomparison.

Laser sources at power levels of the order of 1 mW were scheduled to be used at NIST and some of the other laboratories, which brought about the possibility that the *np*-type photodiodes could be operated in a slightly non-linear region, i.e., become saturated [13,16], at the higher power levels. Applying a reverse bias voltage to the photodiode extends its dynamic range [16,17]. To diminish this probable non-linearity phenomenon, the *np*-type intercomparison radiometers were biased at 4.5 V by three lithium batteries included in the circuit. Applying a reverse bias to a silicon photodiode introduces a large dark current which is considerably noisier than that of the unbiased photodiode. Some precision would therefore be sacrificed in order to obtain a dynamic range of response of the *np*-type intercomparison radiometers that would be comparable to that of *the pn* type [16].

Since lasers were to be used as the radiation source, the radiometers did not have a window covering the silicon photodiode. In the first phase of the intercomparison, the photodiodes were not sealed from possible atmospheric contaminations, but were merely covered by a dust-cap during transport. Although they were all found to be stable at 633 nm under laboratory conditions at NIST, three of the 16 photodiodes, one *pn* type and two *np* types, were found upon remeasurement at NIST to have changed significantly during transport. The *pn* type of photodiode showed a decrease in response at 633 nm of 4% and the two *np* types an increase of 8% and 9.5%. In subsequent rounds of the intercomparison, an attempt was made to more adequately protect the photodiodes by a redesigned cap that sealed off the detector from the atmosphere and by packaging the entire radiometer in a sealed plastic bag containing a desiccant. No changes of such magnitude were observed in the subsequent rounds of the intercomparison. The satisfactory stability of the diodes used in the intercomparison (excluding those that exhibited large changes and were not included in the data analysis) can be assessed from the differences of the before and after NIST measurements. For example, at 633 nm, the average absolute value of these differences was only 0.12%.

The laboratories that received the radiometers suffering the large changes were NRC and NPL. It was also suspected that the radiometers received by PTB might have changed. All three laboratories were invited to repeat their measurements using a different pair of radiometers. In addition, PTB alsorepeated their measurements on the original pair of radiometers. There was no statistically significant difference in the PTB results on either pair of radiometers. Both sets of the PTB measurements are listed in table 1, but only the averages were used in the calculation of the final result. For NRC and NPL, only their second set of measurements on the more stable transfer radiometers are listed in table 1 and used in the analysis of the intercomparison. In the case of the 633-nm measurements at PTB, NRC, and NPL, it was not possible to repeat the NIST measurements after the laboratory’s measurements.

The original plans for the intercomparison called for the radiometers to be measured at NIST both before and after transport to the participating laboratories. Because of difficulties with the argon-ion laser at NIST, it was not possible to perform the first set of NIST measurements at 488 nm. All 633-nm measurements (except for those of PTB, NRC, and NPL as noted above) were performed at NIST before and after transport. For all the laboratories reporting measurements at 488 nm, except NRC and NPL, the NIST measurements were performed after transport. For NPL, the 488 nm NIST measurements were performed before transport; and, for NRC, they were performed both before and after transport. The detailed timetable of the measurements is contained in reference [1]. The lack of consistent before and after NIST measurements is an unfortunate shortcoming of the intercomparison. However, as will be seen below, the results are still remarkably good.

Besides being asked to measure the absolute response of the photodiodes, the laboratories were asked to describe some of the essential features of their measurement process. A summary of the experimental conditions during this intercomparison at each participating laboratory, including NIST, is given in table 1. The uncertainty relative to SI is the laboratory’s estimate of their absolute accuracy with respect to the SI units. The transfer uncertainty is the estimated error incurred in transferring a primary or base measurement to the actual intercomparison measurement. The total measurement uncertainty of a given laboratory is determined from the quadrature sum of these factors and the standard deviation of the measurements reported. The values for the various uncertainties are for one standard deviation for the measurement parameter discussed. The type of optical radiation source used in the intercomparison was either a laser, listed as L in table 1, or a monochromator system, listed as M. Approximate power levels are given in microwatts (W), milliwatts (mW) or nanowatts (nW).

The above experimental conditions listed in table 1 can be summarized as follows. Five laboratories reported using an electrical substitution radiometer as the absolute base of their measurements. Seven laboratories used the predictable quantum efficiency method, originally called the silicon photodiode self-calibration method [18,19]. One laboratory, NML, reported two sets of measurements, that is, they used both types of absolute detectors. They observed no difference in the two techniques at 633 nm and only a 0.1% difference at 488 nm [1]. Their electrical substitution radiometer results are reported in this paper.

Eight of the 11 laboratories that measured the radiometers at 633 nm used a laser at powers ranging from 0.04 to 1.4 mW. The remaining three used either a conventional source and monochromator at a power level as low as 40 nW, or an interpolation from laser-based measurements at lines other than 633 nm. Four of the nine laboratories participating in the 488-nm intercomparison reported using an argon ion laser at power levels ranging from 0.1 to 1.4 mW. Of the five remaining laboratories one reported making measurements at the 20-nW level. The dynamic range of this intercomparison covered nearly five decades of radiant power.

## 3. Results

**Figure 1:**
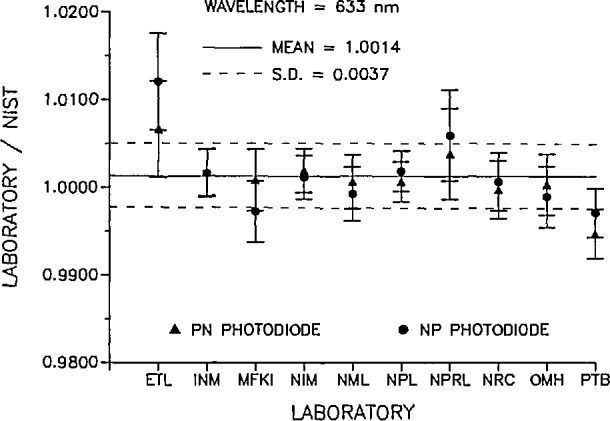
Ratio of participant laboratory spectral response to that determined by NIST at 633 nm; circles indicate *pn*-type detectors and the triangles are *np*-type detectors. The error bars indicate the quadrature summation of the uncertainty relative to SI, the uncertainty of the transfer and the precision of the comparison measurement for each laboratory and the appropriate quantities for NIST. The dashed lines indicate the standard deviation of the measurement for 633 nm.

**Figure 2:**
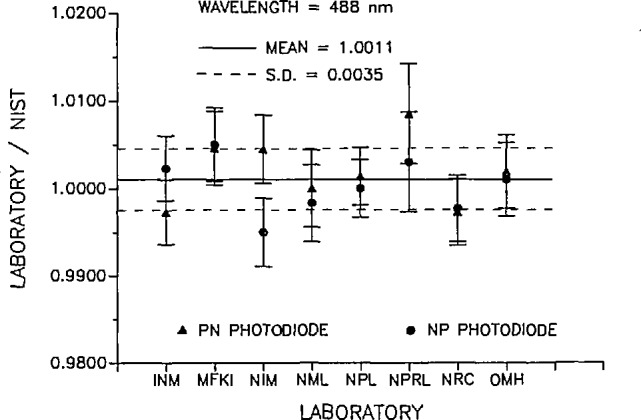
Ratio of the participant laboratory spectral response to that determined by NIST at 488 nm; circles indicate *pn*-type detectors and the triangles are *np*-type detectors. The error bars indicate the quadrature summation of the uncertainty relative to SI, the uncertainty of the transfer and the precision of the comparison measurement for each laboratory and the appropriate quantities for NIST. The dashed lines indicate the standard deviation of the measurements at 488 nm.

The data from each participating laboratory are summarized in tables 2 and 3. Table 2 contains the results of the measurements at 633 nm and table 3 contains the results of the measurements at 488 nm. The results are plotted in figures 1 and 2, respectively. Not all laboratories received the same set of radiometers, so the results are reported as the ratio of the participating laboratory’s measurement with respect to NIST’s measurement of the same radiometer. Since this ratio is near unity, the percent difference in the measurements can easily be obtained as the difference from unity in the reported ratios. This data reduction of the intercomparison results assumes that the measurement uncertainties incurred at NIST are the same for all the radiometers. The random uncertainties in the NIST measurements also contribute to the level of accuracy of the intercomparison. At 633 nm the standard deviation of the NIST measurements for all the radiometers was 0.14%, and at 488 nm, 0.27%. These uncertainties, when combined in quadrature with the uncertainty of NIST with respect to SI and the transfer accuracy, give 0.23% and 0.32% overall accuracy for NIST at 633 and 488 nm, respectively.

Also included in tables 2 and 3 is the average of the (laboratory—NIST) differences in the measured responsivity for each detector type. The uncertainty shown for the average is the standard deviation of the difference with respect to NIST measurements.

Figure 1 is a plot of the ratio of the participant laboratory’s responsivity measurement to that of NIST at 633 nm. The circles indicate the measurements made with the *pn*-type detectors and the triangles are the *np*-type detector measurements. The error bars are the quadrature sum of each laboratory’s stated absolute accuracy, transfer accuracy, and measurement precision for this intercomparison and the appropriate matching quantities for NIST. The dashed lines indicate the standard deviation for all the plotted ratio measurements.

Figure 2 is a plot of the ratio of the participant laboratory’s responsivity measurement to that of NIST at 488 nm. Again the circles indicate *pn*-type detector measurements and the triangles represent *np*-type detector measurements. The error bars indicate the combined NIST and participant laboratory’s accuracy and precision as in figure 1. The dashed lines indicate the standard deviation for all the plotted measurement ratios.

## 4. Conclusions

The interlaboratory measurement of monochromatic radiant power at two wavelengths shows excellent agreement among the participants. The ability of the national standards laboratories to measure highly coherent laser power, or the radiant power from an incoherent source transmitted by a monochromator, is within an overall standard deviation of approximately 0.4%. This result was obtained by two very different techniques for the measurement of absolute radiant power: conventional electrical substitution radiometry and the new predictable quantum efficiency of high quality silicon photodiodes. Furthermore, this level of interlaboratory agreement spans five decades of radiant power.

The interlaboratory agreement demonstrated by this intercomparison was limited in several ways. The overall accuracy of the measurements at the central laboratory limited the comparison to approximately 0.23% at 633 nm and 0.32% at 488 nm. At the time of this intercomparison the effect of humidity on the *pn*-type photodiodes [6] had not been clearly identified; therefore, the radiometers were not designed to avoid this effect. Finally, because of equipment difficulties, the 488-nm measurements could not be performed before shipment from the central laboratory and changes in responsivity went undetected. In spite of these limitations, this intercomparison demonstrates that high accuracy can be achieved by very different radiometric techniques.

The defects in this radiant power intercomparison can be easily avoided in future work. Calibration transfer radiometers for coherent radiation measurements [7] can now be designed to avoid the instabilities caused by humidity [6]. In addition, a measurement precision of 0.03% has been demonstrated for a radiant power measurement at wavelengths of 488 and 633 nm [7]. From the results of this radiant power intercomparison and the improvement in silicon photodiodes and radiometric techniques, we may expect to see properly employed silicon diode detectors lead to improvements in absolute radiometry and photometry in the future.

One general conclusion from this intercomparison arises from the demonstrated agreement between the electrical substitution and silicon self-calibration techniques for measuring radiant power. The silicon self-calibration technique results in a radiometer that is simpler and less expensive than an electrical substitution instrument of comparable accuracy. Although the spectral range of the self-calibration technique is limited to the visible and near visible, coupling it with other nonabsolute radiometers opens the possibility of utilizing its accuracy and ease of use over much of the optical spectrum.

## Figures and Tables

**Table 1:** Summary of uncertainties and experimental conditions

Lab name	Wave length	Absolute base	Uncertainties		(%) Prec	Total	Source	Power
			SI	Trans				
ETL	633	PQE	0.5	0.005	0.04	0.50	L	40 *μ*W
INM	633	PQE	0.12	0.08	0.05	0.15	L	70 *μ*W
	488	PQE	0.12	0.08	0.11	0.18	M	
MFKI	633	PQE	0.15	0.2	0.1	0.27	M	1.5 *μ*W
	488	PQE	0.15	0.2	0.1	0.27	M	1.5 *μ*W
NIM	633	PQE	0.05	0.06	0.06	0.10	L	1 mW
	488	PQE	0.10	0.11	0.15	0.21	L	1 mW
NIST	633	PQE	0.15	0.1	0.14	0.23	L	0.1 to
	488	PQE	0.15	0.1	0.27	0.32	L	0.6 mW
NML	633	ESR	0.05	0.07	0.09	0.12	L	0.5 mW
	488	ESR	0.05	0.07	0.15	0.17	L	1.4 mW
NML^a^	633	PQE	0.1	0.1	0.15	0.21	L	0.5 mW
	488	PQE	0.2	0.1	0.2	0.30	L	1.4 mW
NPL	633	ESR	0.005	0.05	0.02	0.05	L	0.5 mW
	488	ESR	0.005	0.05	0.06	0.08	L	1 mW
NPRL	633	ESR	0.2	0.3	0.3	0.47	M	0.1 to
	488	ESR	0.2	0.3	0.3	0.47	M	200 *μ*W
NRC	633	ESR	0.1	0.1	0.2	0.24	L	0.3 to
	488	ESR	0.1	0.1	0.15	0.21	L	0.8 mW
OMH	633	PQE	0.25	0.06	0.07	0.27	M	40 nW
	488	PQE	0.25	0.06	0.09	0.27	M	20 nW
PTB	633	ESR	0.1	0.1	0.06	0.15	L	0.2 to 1.4 mW

a The PQE based results of NML not used in averages

L=laser source, M=monochromator based source.

Average uncertainty: 633–0.24%; 488–0.25%.

**Table 2:** Absolute responsivity measurement—633 nm

Lab name	Detector type	Lab (A/W)	NIST (A/W)	Lab/NIST	% Uncertainty in ratio
ETL	*pn*	0.4625	0.4594	1.0067	0.55
	*np*	0.4199	0.4149	1.0121	0.55
INM	*pn*	0.4497	0.4489	1.0018	0.27
	*np*	0.4144	0.4137	1.0017	0.27
MFKI	*pn*	0.4494	0.4490	1.0009	0.35
	*np*	0.4123	0.4134	0.9973	0.35
NIM	*pn*	0.4618	0.4609	1.0020	0.25
	*np*	0.4121	0.4116	1.0012	0.25
NML	*pn*	0.4567	0.4564	1.0007	0.31
	*np*	0.4094	0.4097	0.9993	0.31
NPL	*pn*	0.4560	0.4557	1.0007	0.23
	*np*	0.4145	0.4137	1.0019	0.23
NPRL	*pn*	0.4578	0.4560	1.0039	0.52
	*np*	0.4163	0.4138	1.0060	0.52
NRC	*pn*	0.4490	0.4486	0.9998	0.35
	*np*	0.4158	0.4155	1.0007	0.35
OMH	*pn*	0.4492	0.4490	1.0004	0.35
	*np*	0.4130	0.4134	0.9990	0.35
PTB^a^	*pn*	0.4529	0.4552	0.9949	0.27
	*pn*	0.4566	0.4590	0.9948	0.27
	*np*	0.4152	0.4168	0.9962	0.27
	*np*	0.4133	0.4141	0.9981	0.27

a PTB repeated their measurements on both the original and a second pair of silicon photodiodes because of possible detector instabilities. The average of their two measurements was used in the calculations and in figure 1.

Average percent difference (Lab—NIST): *pn—* 0.12±0.30%; *np—* 0.16±0.45%.

Average combined uncertainty in ratio = 0.35%.

**Table 3:** Absolute responsivity measurement—488 nm

Lab name	Detector type	Lab (A/W)	NIST (A/W)	Lab/NIST	% Uncertainty in ratio
INM	*pn*	0.2616	0.2623	0.9973	0.37
	*np*	0.2998	0.2991	1.0023	0.37
MFKI	*pn*	0.2635	0.2623	1.0046	0.42
	*np*	0.3006	0.2991	1.0050	0.42
NIM	*pn*	0.2915	0.2902	1.0045	0.39
	*np*	0.2996	0.3011	0.9950	0.39
NML	*pn*	0.2863	0.2863	1.0000	0.44
	*np*	0.2994	0.3026	0.9983	0.44
NPL	*pn*	0.2827	0.2823	1.0014	0.33
	*np*	0.2994	0.2994	1.0000	0.33
NPRL	*pn*	0.2862	0.2838	1.0085	0.57
	*np*	0.3001	0.2992	1.0030	0.57
NRC	*pn*	0.2613	0.2620	0.9973	0.38
	*np*	0.2976	0.2983	0.9977	0.38
OMH	*pn*	0.2628	0.2623	1.0014	0.42
	*np*	0.2994	0.2991	1.0010	0.42

Average percent difference (Lab—NIST): *pn—* 0.19+0.44%; *np—* 0.03+0.32%.

Average combined uncertainty in ratio=0.42%.

## Footnotes

1 Certain commercial equipment, instruments, or materials are identified in this paper to specify adequately the experimental procedure. Such identification does not imply recommendation or endorsement by the National Institute of Standards and Technology, nor does it imply that the materials or equipment identified are necessarily the best available for the purpose.
